# Terahertz Plasmonic Sensor Based on Metal–Insulator Composite Woven-Wire Mesh

**DOI:** 10.3390/bios12090669

**Published:** 2022-08-23

**Authors:** Ja-Yu Lu, Po-Lun Chen, Borwen You

**Affiliations:** 1 Department of Photonics, National Cheng Kung University, No. 1 University Road, Tainan 70101, Taiwan; 2 Department of Physics, National Changhua University of Education, No. 1 Jinde Road, Changhua 500207, Taiwan; 3 Department of Applied Physics, University of Tsukuba, Tennodai 1-1-1, Tsukuba 305-8573, Ibaraki, Japan

**Keywords:** terahertz, spectroscopy, metamaterial, biomolecular sensor, sensitivity, thickness sensing, refractive index, plasmonic, frequency shift

## Abstract

Terahertz (THz) spectroscopy has been proven as an effective detection means for the label-free and nondestructive sensing of biochemical molecules based on their unique roto-vibrational transitions. However, the conventional THz spectroscopic system is unsuitable for minute material sensing due to its far-field detection scheme, low sample amount, and lack of spectral characteristics, leading to low absorption cross-sections and sensitivity. In this study, a 3D plasmonic structure based on a metal-coated woven-wire mesh (MCWM) was experimentally and numerically demonstrated for sensing trace amounts of analytes combined with THz spectroscopy. Dual sharp spectral features were exhibited in the transmission spectrum, originating from the resonant excitation of THz surface electromagnetic modes via the aperture and periodicity of the MCWM unit cell. According to the finite element simulation, an enhanced and localized surface field was formed at THz resonant frequencies and was concentrated at the metal gaps near the periodic corrugations of the MCWM, resulting in enormous resonant dip shifts caused by the tiny variations in membrane thicknesses and refractive indices. Different types and quantities of analytes, including hydrophilic biopolymer (PAA) membrane, nonuniformly distributed microparticles to mimic macro-biomolecules or cells, and electrolyte salts of PBS, were successfully identified by the MCWM sensor with the best thickness and refractive index sensitivities approaching 8.26 GHz/μm and 547 GHz/RIU, respectively. The demonstrated detection limit of thickness and molecular concentration could respectively achieve nanometer and femtomolar scales in PAA macromolecular detection, surpassing the available metallic mesh devices. The MCWM-based sensing platform presents a rapid, inexpensive, and simple analysis method, potentially paving the way for a new generation of label-free microanalysis sensors.

## 1. Introduction

Sensing trace amounts of biochemical materials is in urgent demand in various applications, such as food safety, illicit substance inspection, environmental pollutant monitoring, and industrial leak detection. Conventional methods for sensing minute materials include electrochemical and optical detection. A semiconductor-based electrochemical device usually needs a certain metal oxide for specific identification and operates under a high temperature with an electric bias for increasing sensitivity. This operation results in temperature-dependent sensitivity and the risk of sparks for explosive material detection [1], which is not suitable for handheld device applications due to security concerns. Optical sensing techniques, including fluorescent labeling [2,3] and spectroscopy [4,5], are popular for molecular identification. Fluorescent labeling is precise for biomedical immunoassays, but it is expensive, time consuming, and photobleaching. It also modifies the intrinsic properties of analytes [2,3]. In contrast, spectroscopy using electromagnetic waves via free space and integrated solid-state configurations, such as dielectric fibers [6,7,8] or slab waveguides, is fast and label-free, but exact light sources from X-rays to microwaves should be selected to achieve noninvasive and nondestructive molecular sensing.

Terahertz (THz) radiation, which lies between visible light and microwaves, possesses low photon energy at a millivolt level, and most nonmetallic barriers can allow the passage of THz radiation with imaging transparency. Many kinds of biochemical molecules, such as proteins [5], nucleic acids [9], and polymers [10], have unique fingerprints in the THz spectral range that arise from intermolecular or intramolecular roto-vibrational transitions. As a result of these fingerprints, THz radiation is capable of label-free, noninvasive, and nondestructive molecular sensing. However, THz spectral sensing is a far-field detection method in its conventional form. For a tiny analyte, a small absorption cross-section exists between the THz field and the target analytes, which consequently decreases the output signals of transmittance or reflectance. Therefore, a signal enhancer needs to be combined with THz spectroscopy for the highly sensitive detection of a minute sample.

Two approaches to increase the sample interaction strength with THz radiation are considered efficient strategies for enhancing weak output signals. One is to increase the concentration of adsorbed or infiltrated analytes on the device, such as by increasing the number of ligands and the area-to-volume ratio of the device. The other is to strengthen the electromagnetic intensity concentrated on the active sites of the device based on the surface plasmon phenomenon, such as in optical surface-enhanced Raman spectroscopy [11,12] and surface-enhanced absorption spectroscopy [12,13], through various metamaterials and plasmonic or photonic structures [14,15]. A metamaterial is made of thousands of subwavelength-unit elements (atoms) with a periodic arrangement. The artificial material can be created with a complexed dielectric constant or optical properties that do not exist in nature, such as a negative refractive index [16], superlensing [17], and optical cloaking [18]. The transmitted or reflected electromagnetic property of the metamaterial can be manipulated at will by engineering its geometry parameters to realize various functionalities, such as modulators, filters, and resonators [19]. In addition, the spectral property of the plasmonic metamaterial is geometry-dependent and highly sensitive to the surroundings near the hot spot [12], benefiting sensing applications. On the basis of the suitable pattern design and arrangement of unit cells, the plasmonic artificial structure can overcome the optical diffraction limit to effectively couple the impinging electromagnetic radiation into an enhanced and localized electromagnetic field on a deep-subwavelength scale, greatly increasing the molecular absorption cross-section and realizing the ultrasmall-quantity detection of molecules.

Metallic mesh devices (MMDs) [20,21,22,23,24,25,26] are one example of plasmonic metamaterials with the unique property of extraordinary transmission (EOT) [26,27,28] at a certain band. Their collective resonant transmission is caused by the excitation of surface plasmon polaritons (SPPs). Apart from EOT resonant peaks, a Fano-like spectral dip [20,21,24,25] can also form near the EOT peak at an oblique incidence. Strongly enhanced field intensities are localized within the MMD openings at resonant frequencies. The characteristic frequencies of the metallic mesh at resonant peaks and dips are associated with the geometric parameters of the MMD and the refractive indices of the media surrounding the MMD openings [20,21,22,23,24,25,26]. The resonant frequencies can be tuned in a wide spectral range from visible to the THz regime by adjusting the opening sizes and substrate indices of the MMD [21,23,26]. The wide spectral tunability of MMDs enables the decay length and resonant frequencies of the evanescent field to match the target size and fingerprints of the target analyte, respectively, in order to increase the absorption cross-section and detection selectivity. The sensitivity of the characteristic frequencies to complex refractive indices causes explicit frequency shifts or amplitude variations in the transmission or reflection spectra after an MMD is loaded with analytes. This increases the identification capability for wide varieties of molecules, such as proteins [20], bacteria, cells, and antibiotics [25] in aqueous solutions or dry forms. For example, the highest molar sensitivity of the MMD sensor for protein detection has been experimentally demonstrated to reach around 10 femto-mole/mm^2^ (i.e., 1 ng/mm^2^) [20], which operates in the mid-IR frequency range with an MMD resonant dip frequency of 100 THz. For thin-film sensing, the limit of thickness detection using an MMD is around 100 nm at a dip frequency of 10 THz, corresponding to λ/300 [21]. However, for the two sensing platforms of MMDs, an oblique wave incidence is necessary to produce a sharp dip as a sensing probe. This angle-tilting operation has a very low tolerance for repetition at the same dip frequency. In addition, the planar type of metallic hole array (MHA) has limited sensitivity unless the probing frequency of the MMD is raised to that of an infrared ray to improve the sensing performance.

In this paper, a 3D plasmonic structure made of a plastic woven-wire mesh with a one-sided metallic coating is proposed for the label-free sensing of trace amounts of analytes in the THz regime. The spectral responses and electric field distributions of the metal-coated woven-wire meshes (MCWMs) were experimentally and numerically characterized. An enhanced and localized surface field was formed at THz resonant frequencies and was concentrated on the fine metallic structures of the plasmonic device, promoting sensitivity to tiny variations in the nearby environment of the MCWM. The optimum structure and preferable characteristic frequency of the MCWM sensor were determined via thin-film sensing under normal incidence. Dielectric membranes made of hydrophilic macropolymer molecules with various thicknesses and refractive indices were deposited on the metal surface of the MCWM to explore the sensing mechanism. Instead of attaching nano- or microthick thin films onto the peripheral surface of the MCWM, we used a deposition process based on a drop-and-dry method, which caused the viscous analyte solution to completely infiltrate the tiny interstices of the MCWM structure, so that it effectively interacted with the localized plasmonic fields. For future biological sensing applications, nonuniformly distributed samples, including electrolyte salts of phosphate-buffered saline (PBS) and polymeric microspheres, were further prepared and tested by utilizing the MCWMs. The polymeric microspheres were used to mimic macro-biomolecules or cells in molecular sensing. PBS is the most commonly used buffer in cell culturing, DNA detection, and many biological experiments. The accurate and quantitative detection of the electrolyte salt concentration in PBS is important to ensure its PH-adjustment and isotonicity functions. The quantitative analyses of the sensitivity and limit of detection (LOD) were characterized in terms of sample thickness, refractive index, weight, and molar density. In the discussion section, the sensing performance of the MCWM-based plasmonic structure is compared with that of metamaterial- and metal-hole-array-based devices.

## 2. Materials and Methods

### 2.1. MCWM Materials and Sensing Samples

**Figure 1:**
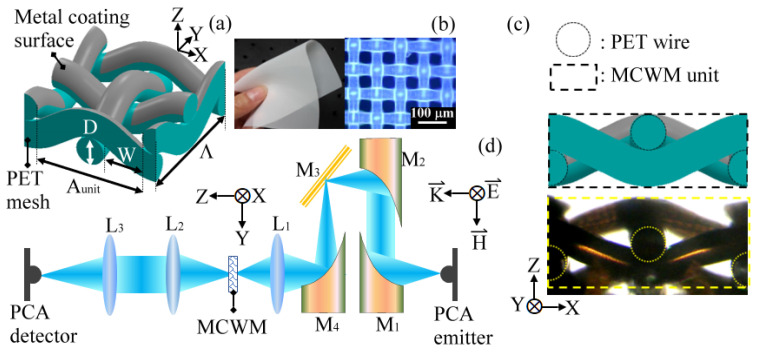
(**a**) Unit structure of MCWM (a 3D-view drawing); (**b**) macroscopic and microscopic photographs of a PET mesh; (**c**) side views of a schematic drawing and a microscopic photograph of an MCWM unit structure; (**d**) configuration of the reading unit (i.e., the THz-TDS system) for a MCWM sensing device. L_1_–L_3_: plastic lenses; M_1_–M_4_: metal mirrors; PCA: photoconductive antenna.

The 3D plasmonic structure was composed of a woven-wire-mesh-based plastic substrate with periodic metal segments on one side of the woven-wire surface. Figure 1a presents a schematic sketch of a single periodic unit of the MCWM-based plasmonic structure. The plastic woven-wire mesh was formed of transversely interwoven polyethylene terephthalate (PET) polymeric wires. As shown in Figure 1b, the plastic substrate was flexible and comprised periodically spaced square apertures in the square lattice. A metallization layer made of 200 nm thick aluminum (Al) with a conductivity of 0.77 MS/cm was deposited on one side of the PET woven-wire mesh through the electron beam evaporation method to form the MCWM device (Figure 1a). The metallic surface of the MCWM device was discontinuous due to the intersection of the PET woven wires and was composed of multiple metal segments periodically arranged on one side of the plastic mesh substrate. Figure 1c shows side views of a schematic drawing and a microscopic photograph of the MCWM structure, displaying the MCWM morphology of transversely interwoven PET wires in contrast to that of a planar MHA. The PET woven-wire mesh substrates were purchased from SEFAR AG (Heiden city, Switzerland, Model: SAFER PET 1500). The geometric parameters of the PET mesh membranes used in this experiment are listed in Table 1, including the square pore width (*W*), wire width (*D*), unit-cell period (*Λ*), and open ratio. The lattice period (i.e., the unit-cell period or the pitch) of an MCWM is determined from the periodically sinusoidal corrugations of the woven wires, which are indicated as *Λ* in Figure 1a and defined as *Λ* = 2 (*W* + *D*) in quantity. In contrast to planar MHAs, MCWMs have not only a square pore width (*W*) but also a unit-cell pore width (*A_unit_*) in a 2D array (i.e., the X–Y plane in Figure 1a), whose dimensions are defined as *A_unit_* = *2 W + D* and are specifically approximate to the maximum length of a continuous metal-coated wire in a unit cell. The open ratio indicates the area percentage of air and is defined as *W*^2^/(*W* + *D*)^2^, which is uniform in the macroscopic version for a dielectric substrate. The PET polymer retains good mechanical properties at temperatures of up to 175 °C due to its high melting point (ca. 255 °C) and glass transition temperature (ca. 70 °C) [29]. The linear thermal expansion coefficient of PET is about 6 × 10^−5^ per °C [30]. For a 1 mm thick PET film, the thickness expansion is about 0.06 μm per °C, which is much smaller than the THz wavelength and decay length of the THz plasmonic surface field on the MCWMs. The geometric variation of the MCWMs induced by temperature could thus be ignored. In addition, variations in the temperature-dependent THz refractive index or absorption in plastic and polymer materials have not been observed [31,32,33]. The ambient temperature in the MCWM sensing experiments was controlled at 27 ± 0.5 °C.

In the sensing experiments, all the target samples were deposited on the metal surface of the MCWMs via a drop-and-dry process. For thin-film sensing, various thicknesses of dried polyacrylic acid (PAA) polymer films were utilized to calibrate the thickness sensitivity, and their fabrication process was as follows. A 1 wt% PAA aqueous solution was prepared by dissolving the PAA powders (Carbopol 940, cP 45,000–80,000, Lubrizol Advanced Materials, OH, USA) in reverse-osmosis water. This highly viscous PAA aqueous solution was then dropped onto the Al metal surface of the MCWM using a micropipette. A wet PAA membrane was thus formed with a diameter of around 6 mm. The wet PAA membrane was dried by a fan for three hours until its weight was stable. Multiple layers of dried PAA films were fabricated by repeating the drop-and-dry process described above to form various thicknesses of thin-film analytes for thickness sensing. PAA is a hydrophilic and highly absorbent polymer that has been widely applied in biomaterials due to its high solubility, good bioadhesion, and biodegradability [34]. Thin-film fabrication based on the drop-and-dry method ensured that the polymer material infiltrated the aperture arrays and filled the tiny interstices of the sensing chip. For refractive index sensing, different quantities of lactose powders were dissolved in 1 wt% PAA aqueous solutions to produce lactose-doped PAA films with a series of doping concentrations that corresponded to different refractive indices. Furthermore, the hydrophilic PAA membranes could be easily removed via a dipping process in water without destroying the metallic coating surface and the morphology of the MCWM. The transmission spectrum of the blank MCWM was repeatable after each water washing, which benefitted the device’s reusability. However, different MCWM devices, even when they had the same geometry, e.g., the small-pore MCWMs specified in Table 1, were used in different sensing experiments and still showed slight differences of around ±10 GHz in the spectral features due to the issues of structural uniformity. This certainly did not influence the sensing or identification results among the analytes, because the transmittance spectra of the blank MCWMs were simply used as reference signals to be normalized and to obtain analyte-induced spectral shifts.

In the experiment involving the sensing of nonuniformly distributed analytes, two samples were used, namely polyethylene (PE) microspheres and electrolyte salts. The PE microspheres (Sigma-Aldrich, Taipei, Taiwan, Product No. 434272) with particle diameters of 34–50 μm were mixed in 1 wt% PAA aqueous solution with a series of doping concentrations and deposited on the sensing device via the drop-and-dry process. The dried PAA matrix could effectively fix the PE microparticles on the MCWM sensing chip. Different quantities of dried electrolyte salts were prepared from 10-times-concentrated Dulbecco’s phosphate-buffered saline (10X DPBS, PBL05, Caisson Labs, Smithfield, UT, USA) with different concentrations diluted in deionized water. Various volume concentrations of the diluted DPBS solutions with a definite liquid volume were dropped onto the MCWM sensing chip and then dried naturally to form different amounts of nonuniform salt grains. The DPBS solution used in this experiment contained KCl, KH_2_PO_4_, NaCl, and Na_2_HPO_4_, with NaCl being the dominant component.

### 2.2. Experimental Setup and Spectra Acquisition

The spectral properties of the MCWMs were characterized using a transmission-type THz time-domain spectroscopy (THz-TDS) system. In the sensing experiment, the reading unit of the MCWM sensing device was also the THz-TDS system, operated at room temperature and including a femtosecond pulse laser, a pair of photoconductive antennas (PCAs) for the THz wave emitter and detector, and a motional stage for an optical delay line [12]. As illustrated in Figure 1d, a pair of LT-GaAs-based PCAs were optically excited by a mode-locked Ti:sapphire laser, i.e., a femtosecond pulse laser, with a central wavelength of 800 nm, a pulse width of 100 fs, and a repetition rate of 82 MHz for THz wave emission and detection. The PCA-based THz emitter and detector were both equipped with an objective lens for focusing the free-running femtosecond laser pulse on the 5 μm wide electrode gaps of the PCAs. Achieving the appropriate alignment between the laser beam spot and the radiation location on the PCAs required confocal imaging skills to realize the highly efficient radiation and detection of THz pulses through the correct laser beam spots. Furthermore, the flight time between the THz and laser pulses were regulated via an optical delay line controlled by a motional stage to ensure that they arrived at the PCA-based detector at the same time. The highest THz pulse amplitude was therefore obtained due to the equal optical path of the THz and laser pulses at the PCA-based detector [12].

As shown Figure 1d, the generated THz pulse was collimated by a parabolic mirror and focused on the MCWM chip by a plastic lens with an effective focal length of 25 mm (L_1_ in Figure 1d). The metal surface side of the MCWM chip was illuminated by a normally incident THz wave with the electric field parallel to the x-axis (Figure 1d). The THz signal transmitted through the rear plastic surface of the MCWM was then collected by a pair of plastic lenses with a focal length of 50 mm (L_2_ and L_3_ in Figure 1d) and coupled into a PCA detector for a THz waveform measurement. The time-domain THz waveform could be obtained by repeatedly scanning the optical delay line. A function generator synchronously modulated the PCA emitter and a lock-in amplifier with a frequency of 2 KHz and a peak-to-peak voltage of 4 V. The signal-to-noise ratio (SNR) of the THz waveform was thus raised. Through the fast Fourier transformation of a measured THz waveform, the THz power spectrum of 0.1–1.0 THz could be obtained, and the spectral peak was determined at 0.3 THz with a power SNR of up to 10^5^. The equipment cost for a femtosecond-laser-driven THz-TDS system is relatively high compared with visible or infrared spectroscopy. However, the THz-TDS system is advantageous, as it can easily acquire both the amplitude and phase of the transmitted/reflected THz pulse for a single waveform scan. The analyte information, including the real and imaginary parts of the optical constant, was thus obtained without using the complex Kramers–Kronig relation [35]. A frequency-tunable CW THz system based on a Gunn oscillator, frequency multipliers, a Golay cell, or a Schottky diode [36] could also be utilized as a low-cost reading unit to replace the THz-TDS system in this experiment. 

For the spectral characterization of the MCWMs, the THz transmittance spectra were acquired from the transmitted THz power spectra of the MCWMs and their blank PET mesh substrates. The spectral transmittance of the MCWMs was obtained by comparing the transmitted power spectra of the PET woven-wire meshes with and without Al-metal coating layers. Thus, the impact of the plastic substrate transmittance on the measured results of the MCWMs did not need to be considered, including the material absorption of the PET wire and the scattering loss due to the surface roughness. The transmittance of the MCWMs, *T_MCWM_*, was quantitatively defined as *T_MCWM_* = *P_Al+PET mesh_*/*P_PET mesh_*, where *P_Al+PET mesh_* and *P_PET mesh_* are the transmitted powers of an MCWM and its PET mesh substrate. For the sensing performance of the MCWMs, the transmittance of the analyte-loaded MCWMs (*T_sample_*) could be derived from this relation, *T_sample_* = *P_sample+Al+PET mesh_*/*P_PET mesh_*, where *P_sample+Al+PET mesh_* represents the transmitted power of a sample-loaded MCWM. Each spectrum collected in the study was an average of three measurements for each sample. 

## 3. Results and Discussion

### 3.1. Characterization of THz Electromagnetic Response of MCWM

#### 3.1.1. Spectral Characteristics

**Figure 2:**
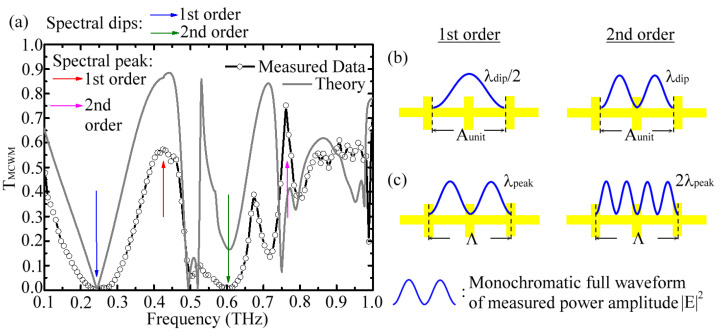
(**a**) Experimental and simulated transmittance spectra of 249 μm *W* MCWM; schematic diagrams of the characterized spectral (**b**) dip and (**c**) peak wavelengths relating to *A_unit_* and *Λ* of the MCWM, respectively.

Figure 2a shows the measured and simulated transmittance (*T_MCWM_*) spectra of the 249 μm *W* MCWM. The numerical simulations of our study were conducted based on the finite element method (FEM) [18] and are shown as a gray curve in Figure 2a. For FEM modeling, periodic boundary conditions were applied to mimic a 2D infinite structure. The unit cell structure of the MCWM in the numerical simulation is illustrated in Figure 1a. All the Al metal segments were modeled with a perfect electric conductor and were uniformly deposited on the PET woven-wire surfaces with a 200 nm thickness. The broadband THz plane waves (0.1–1.0 THz) with linear polarization, as shown in Figure 1d, were normally illuminated on the metal-coated side of the MCWM in both the FEM model and the THz-TDS measurement experiment. The orientation of the square aperture arrays of the MCWM, i.e., the azimuthal angle along the z-axis in Figure 1d, was insensitive to the incident polarization of THz waves based on the measured transmission spectrum.

In Figure 2a, two orders of spectral dips and peaks existed within the 0.1–1.0 THz frequency range. The first-order dip/peak of the MCWMs had a longer wavelength and was located in a lower THz frequency range than the second-order dip/peak of the MCWMs. The numerically simulated spectra reasonably agreed with the measured results, especially at the characteristic frequencies. The measured (simulated) characteristic wavelengths of the 249 μm *W* MCWM in Figure 2a were 1.248 mm (1.227 mm), 0.706 mm (0.682 mm), 0.602 mm (0.607 mm), and 0.394 mm (0.420 mm), corresponding to the first transmission dip, the first transmission peak, the second transmission dip, and the second transmission peak, respectively.

For the 249 μm *W* MCWM, we further associated the dip wavelengths with the geometric parameters and found a strong correlation between the measured spectral dip wavelengths (*λ_dip_*) and its unit-cell pore width, *A_unit_* ~ 0.618 mm. One half of the first-order dip wavelength (*λ_dip_* ~1.248 mm) nearly matched the *A_unit_* value, and the wavelength of the second-order spectral dip (*λ_dip_*~0.602 mm), i.e., two half wavelengths or a full wavelength, approximately matched the *A_unit_* value. Studies have shown that the spectral features of 2D planar MHAs, such as EOT [26,27,28] and Fano-like resonances [20,21,24,25], strongly rely on the surface electromagnetic (EM) modes excited on the metal hole surface. On the basis of the theory of THz wave resonance within metal cavities [37], integral multiples of half-wavelengths for the input THz waves should have been approximate to the *A_unit_* value (0.618 mm) of the 249 μm *W* MCWM (Figure 1a), which was consistent with the measured and simulated results of the spectral dips in Figure 2a. The corresponding relation between the *A_unit_* value and the characterized dip wavelength (*λ_dip_*) was thus diagramed in Figure 2b. This finding revealed that, for the 249 μm *W* MCWM, the surface EM modes of the transmission dip frequencies originated from resonantly coupling the input THz waves into the surface modes via the periodically arranged apertures of the unit cells (*A_unit_*).

The transmission mechanism of the resonant peaks for an MCWM is associated with the EOT phenomenon of a planar MHA device and is described below. For an MMD unit, the approximate resonant wavelength of the transmission peak results from EOT resonance, obeying Equation (1):

(1) $$\lambda_{r}=\frac{p\sqrt{\varepsilon_{d}}}{\sqrt{i^{2}+j^{2}}}$$

This formula is derived from so-called Wood’s anomalies [26]. The wavelength of the resonant peak *λ_r_* is determined by the MMD lattice period, *p*; the permittivity of the dielectric adjacent to the conductor, *ε_d_*; and the mode indices *i* and *j*, which are spatial harmonics belonging to integers. The strong transmission peak linked to EOT resonance results from resonantly coupling the incident wave via the periodicity of the hole array on the input faces of an MHA into the surface EM mode. The surface plasmon polariton (SPP)-like EM energy propagates along the metal–dielectric surface and couples in a leaky manner with the MHA output face through the holes [38]. This phenomenon is also called the leaky-wave property of MHA. According to Equation (1), the EOT resonance wavelengths *λ_r_* of the 249 μm *W* MCWM were theoretically calculated as 0.708 and 0.394 mm for the first- and second-order peaks, respectively, where the *p* values of Equation (1) were individually substituted by the *Λ* and *Λ*/2 values of the 249 μm *W* MCWM (Figure 1a). The corresponding *ε_d_* values for each *λ_r_* were determined from the effective medium theory with the factors of the open ratio (Table 1) and the dielectric constants of pure PET and air. That is, the EOT resonant wavelength shifted to a relatively low frequency range according to Equation (1) when an effective refractive index of the dielectric mesh substrate, represented by $\sqrt{\varepsilon_{d}}$, increased with a low open ratio. The spatial harmonic parameters of (i, j) were denoted as (1, 1) for this MCWM *λ_r_* estimation. The theoretically calculated values of the MCWM *λ_r_* were approximate to the measured values and the numerical simulation of the first and second wavelength peaks (Figure 2a). Therefore, the two orders of the 249 μm *W* MCWM spectral peaks, as shown in Figure 2a, had an evident correlation with the period of an MCWM unit cell (i.e., a *Λ* or 2 (*W + D*) value), which is a general characteristic of EOT resonance [26,27,28]. The relation between the *Λ* value and the characterized peak wavelength (*λ_peak_*) is diagramed in Figure 2c. This result revealed that the first and second resonant peaks of the MCWMs represented the SPP-like surface EM modes that were excited at the metal–PET interface, and this effect arose from the resonant coupling of incident THz waves via the different lattice periods of the aperture arrays. This means that the first EOT peaks were generated based on the periodicity of the unit-cell arrays, i.e., the period of the unit cell *Λ*, with a spatial harmonic of (1, 1). The second-order spectral peaks were generated with the same spatial mode of (1, 1) but were coupled by the periodicity of a small aperture, corresponding to the *Λ*/2 period (Figure 1a).

#### 3.1.2. Model Field Performance

**Figure 3:**
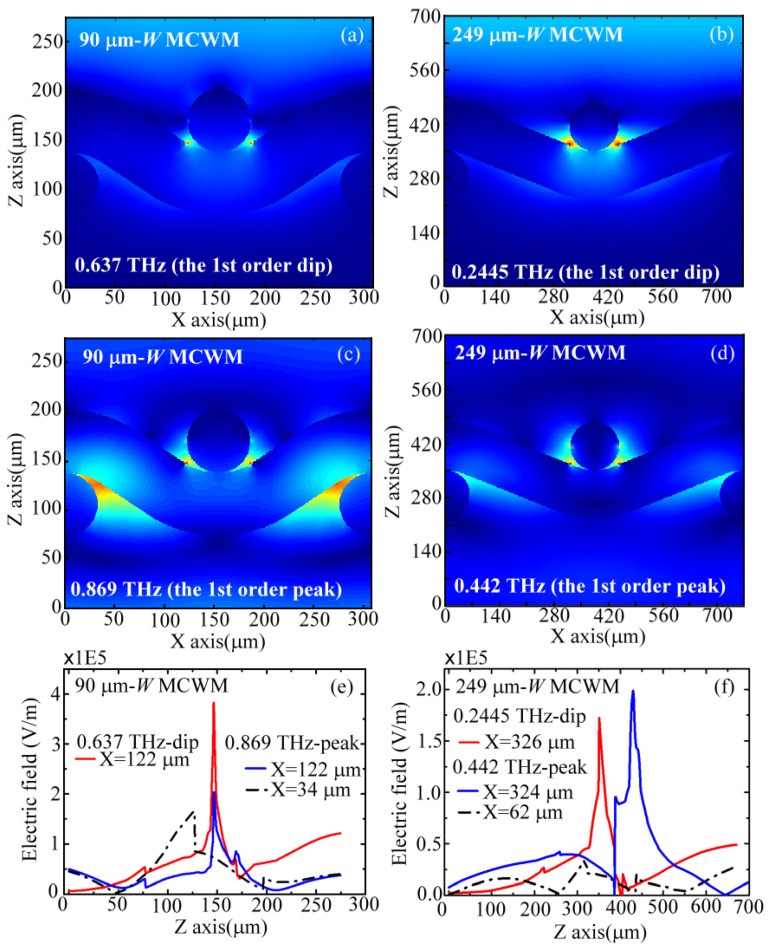
Simulated (**a**–**d**) X–Z lateral modal profiles and (**e**,**f**) *Z*-axial electric field distributions of 90 and 249 μm *W* MCWMs at the first-order spectral dips and peaks.

On the basis of the FEM calculation method, Figure 3a–d depict a cross-section of the simulated electric-field (E-field) distributions within the unit cells at the first transmission dip and peak frequencies for the 90 and 249 μm *W* MCWMs. The E-field modal profiles are presented in *X*–*Z* lateral planes, which are located at the *Y*-axial center of an MCWM unit cell (Figure 1a). The upper and lower sides along the Z-axis are the Al metal-coated surface and the pure PET sides of the MCWMs, for receiving and outputting THz waves, respectively. Figure 3a,b illustrate the E-field distributions of the 90 and 249 μm *W* MCWMs at the first-order spectral dip frequencies of 0.637 and 0.2445 THz, respectively. The simulated E-field distributions showed that the input THz waves at the spectral dip frequencies were strongly concentrated at the metal segment edges of the *X*-axial woven wires. They also demonstrated that the surface EM modes at the first order of MCWM resonance had high confinement without forward propagation. Moreover, much less field energy could be found inside the openings of the unit cell and on the bottom (PET) surface of the MCWM (Figure 3a,b). Figure 3c,d show the FEM simulation results of the E-field distributions at the first-order transmission peaks, which were 0.869 and 0.442 THz for the 90 and 249 μm *W* MCWMs, respectively. The THz waves were not only distributed on the input metal surfaces of the MCWM but were also transmitted through the openings of the unit cells, finally arriving at the pure PET output sides of the MCWM. 

To quantitatively compare the local THz field strengths at the dip and peak frequencies along the *Z*-axis of the MCWM (i.e., the propagation axis), one-dimensional (1D) E-field distributions of the 90 and 249 μm *W* MCWMs were plotted at specific X positions, as shown in Figure 3e,f, respectively. Figure 3e illustrates the 1D E-field distributions of the 90 μm *W* MCWM at the metal segment edge position (*X* = 122 μm), indicating the highest electric field strengths *(E_peak_*) of the spectral dip and peak frequencies, i.e., 0.637 and 0.869 THz, both located at *Z* = 146 μm. However, the *E_peak_*-strength of the dip frequency (red curve) was two-fold higher than that of the peak frequency (blue curve). In contrast, for the E-field distributions of the 249 μm *W* MCWM (Figure 3f) at the metal segment edge position (*X*~325 μm), the *E_peak_* value of the 0.442 THz peak frequency (blue curve) was slightly higher than that of the 0.2445 THz dip frequency (red curve). Figure 3e,f show that enlarging the *W* dimensions of the MCWMs from 90 μm to 249 μm decreased the field concentration of THz surface EM modes at the metal segment edges of the input surfaces. For the 1D modal pattern results of the 90 and 249 μm *W* MCWMs, as shown in Figure 3e,f, the highest field strengths at the PET dielectric sides of the output surfaces (black curve) were considerably weaker than those on the input metal surfaces (red and blue curves), where the locations of the *X*-axis were considered to be at 34 and 62 μm for 0.869 and 0.442 THz, respectively. This intensity distribution of the surface EM field on the metal and pure PET surfaces determined the active areas for sample loading to achieve efficient sensing applications.

### 3.2. Sensing Performance of MCWMs

To investigate the spectral responses of the transmission peaks and dips for analytes with different thicknesses and refractive indices deposited on the MCWM-based sensing chip, a polymeric thin film made of PAA was chosen as the standard sample in the sensing experiment. The sensitivity calibration and demonstration of the simulated surface EM modal field are shown in Figure 3a–d. To quantitatively compare the sensitivities of thin-film detection using MCWM devices with different geometric sizes, the 249 and 90 μm *W* MCWMs were utilized as the sensing chips in the experiments and theoretical simulations to observe their transmission spectral responses at the first spectral dips and peaks. The quantitative analysis of the thin-film detection sensitivities could thus be conducted based on the *W* aperture size and the sensing dip/peak frequencies. 

#### 3.2.1. Thin-Film Sensing: Thickness Detection

**Figure 4:**
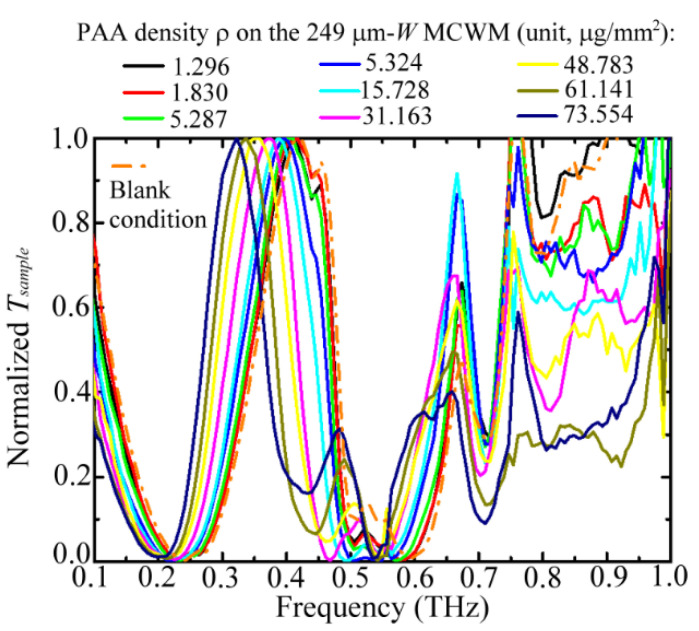
Normalized transmittance of a 249 μm *W* MCWM loaded with different weight densities of PAA membranes for thickness detection experiment.

Figure 4 illustrates the spectral variation of a 249 μm *W* MCWM for nine different PAA membranes; the corresponding transmittance values at the first-order peaks were normalized to focus on their spectral shift effect. The various weight densities of the PAA membranes (*ρ*) in the figure represent different physical thicknesses deposited on the MCWM. The measured results indicated that the spectral shifts increased with the PAA densities for the first-order dip and peak frequencies, but the frequency shift range of the second-order dip was comparatively small for sensing the same PAA membranes. Furthermore, the spectral positions of the second-order peak frequencies were almost stationary as the PAA membrane densities increased. This finding revealed that the traditional EOT resonance, associated with the second-order peak, did not show sensitivity to the dielectric environment through spectral shifts, and only the transmitted intensity of the EOT was modified due to the sample material loss. A sensing mechanism based on the intensity interrogation of the EOT resonance has been demonstrated in various sensing applications [22,24]. In this study, we merely investigated the spectral-shift characteristics of MCWMs at the first-order dip and peak frequencies to identify sample molecular quantities and categories.

**Figure 5:**
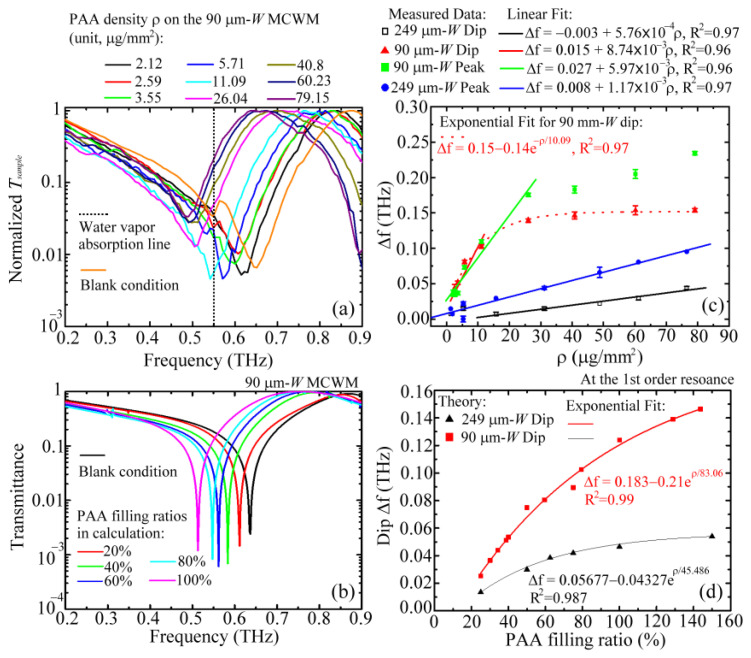
(**a**) Normalized transmittance of a 90 μm *W* MCWM loaded with different weight densities of PAA membranes for thickness detection experiment; (**b**) calculated transmittance of a 90 μm *W* MCWM with different filling ratios of PAA membranes; (**c**) relationship between spectral shift and PAA molecular density for the first spectral dips and peaks of 90 and 249 μm *W* MCWMs; (**d**) calculated spectral dip shifts at the first-order resonance for the 90 and 249 μm *W* MCWMs.

Figure 5a shows the measured transmission spectra of the 90 μm *W* MCWM loaded with nine different weight densities of PAA membranes compared with that under blank conditions (i.e., orange curve). The dip and peak frequencies of the blank 90 μm *W* MCWM were obviously higher than those of the 249 μm *W* MCWM (Figure 2) due to the smaller structural dimensions (Table 1). In terms of the spectral response for thickness sensing, the first-order transmission dip and peak were gradually redshifted in the spectral range of 0.2–0.9 THz as the densities of the PAA membrane increased. For the curves under blank and low-density conditions (*ρ* = 0, 2.59 and 5.71 μg/mm^2^), a fluctuating dip in the normalized *T_sample_*, resulting from the discrepancy in the power measurement for the *P_sample+Al+PET mesh_* and *P_PET mesh_* values, was exhibited at around 0.55 THz, which is an absorption-line frequency of ambient water vapor [39]. Furthermore, considering the spectral resolution of 14 GHz, in Figure 5a, the fluctuating spectral range of the three *T_sample_* curves was exactly 0.549 ± 0.014 THz. Compared to the other water vapor absorption line at 0.75 THz [39], the fluctuating dip did not occur due to sufficiently high power transmission through the 90 μm *W* MCWM, whose transmittance was above 0.1. The normalized *T_sample_* values at the first dip obviously rose as the PAA density increased. However, the resulting Q factors of the first dip of the 90 μm *W* MCWM for sensing each PAA density were certainly sustained without obvious degradation.

The corresponding transmission spectra of the 90 μm *W* MCWM simulated using the FEM method are illustrated in Figure 5b, which shows the first-order dip and peak frequencies for the infiltration of different analyte quantities on the sensing chip. The target PAA membranes are individually presented with different PAA filling ratios (20–100%), which represent various dielectric ratios for the uniform occupation of the 128 μm structural depth of a 90 μm *W* aperture (Table 1). The dielectric membrane thickness in the FEM calculation was determined from the filling ratio of a pore depth, corresponding to the weight density of a PPA membrane in the experiment (Figure 5a). The spectral shifts of the measured (Figure 5a) and simulated (Figure 5b) transmission spectra had the same trend for sensing different thicknesses of PAA membranes. The comparison demonstrated that the infiltration of a dielectric analyte inside the MCWM apertures, i.e., the *W* wide pores in Figure 1, caused an evident spectral shift. A greater sample filling depth within the MCWM pores, representing a greater membrane thickness, resulted in an increased optical path length (OPL) and, thus, a more pronounced spectral shift relative to the spectral dip and peak of the blank MCWM. The experiments also proved that the planar attachment of a 25 μm thick PE film on the 249 μm *W* MCWM prevented any spectral shift of the first-order dip and peak. The sensing feasibility and mechanism of using an MCWM to recognize various membrane thicknesses were experimentally and numerically verified. The infiltration of the dielectric material inside the pores was the criterion for successful identification.

Figure 5c shows the measured frequency shifts ∆*f* of the first-order spectral dip and peak, relative to the individual spectral positions of the blank 249 and 90 μm *W* MCWMs, for the deposition of different quantities of PAA membranes. While the quantity of PAA molecules gradually increased in the pores of the 249 μm *W* MCWM, the ∆*f* values of the peak and dip slowly increased. That is, the dip and peak ∆*f* values were linearly proportional to the PAA membrane density *ρ*. The ∆*f* values of the peak were larger than those of the dip for *ρ* values above 10 μg/mm^2^; conversely, the dip and peak frequency waves had comparable ∆*f* values for *ρ* values lower than 10 μg/mm^2^. For the 90 μm *W* MCWM, the ∆*f* values of the dip and peak both rapidly increased and were linearly proportional to the sample densities before saturation. The MCWM sensitivity for detecting PAA membrane thicknesses could be characterized by the slopes of the linear fitting curves, which are shown in Figure 5c. The dip frequency wave of the 90 μm *W* MCWM had the highest slope value, denoted as *S_d,90_*. The slope of the 90 μm *W* MCWM peak wave, denoted as *S_p,90_*, was obviously higher than the slope values of the 249 μm *W* MCWM dip and peak waves, respectively denoted as *S_d,249_* and *S_p,249_*, but slightly lower than the *S_d,90_* value. That is, the sequence order of membrane thickness sensitivity was *S_d,90_* ≳ *S_p,90_* > *S_p,249_* > *S_d,249_*. The THz surface EM mode of the first-order resonance, corresponding to the 90 μm *W* MCWM dip wave, had the best thickness sensitivity because it had the greatest field–analyte interaction strength based on the most confined modal profile and the highest *E_peak_* within a near-field range, as shown by the red curve in Figure 3e.

Figure 5d illustrates the simulated ∆*f* values of the first dip frequencies for the 249 and 90 μm *W* MCWMs under a series of PAA membrane filling ratios. The dip ∆*f* values of the 90 μm *W* MCWM were obviously greater than those of the 249 μm *W* MCWM for all the PAA membrane filling ratios. That is, the THz surface EM mode of the first-order dip wave for the small *A_unit_* size (90 μm *W* MCWM) had a larger spectral shift Δ*f* than for the large *A_unit_* size (249 μm *W* MCWM). The former device had a denser surface field energy concentration within a shorter near-field range, achieving a larger field–analyte interactive strength compared with the latter device (Figure 3e,f). The simulated dip Δ*f* values increased with the sample amounts, which reasonably agreed with the measured results in Figure 5c. The theoretical spectral responses were gradually saturated with the increased membrane filling ratios and were well-fitted by the exponential curves with a coefficient of determination above 0.98 (R^2^ > 0.98). However, each filling ratio for the two different aperture sizes of the MCWMs did not represent the same PAA quantity inside the apertures of the two MCWMs. Therefore, the saturation behavior of the simulated curves in Figure 5d was not exactly consistent with that of the measurements presented in Figure 5c.

For the same PAA membrane infiltration quantity in the 90 and 249 μm *W* MCWMs (Figure 5c), the small apertures of the 90 μm *W* MCWM reached the saturated spectral shift first due to the highly confined modal profiles at both the dip and peak frequencies (Figure 3e). For the 249 μm *W* MCWM, the distinctly large modal profiles at the corresponding dip and peak frequencies (Figure 3f) were still not fully covered by the maximum PAA density at around 80 μg/mm^2^ because of the large apertures and, therefore, it did not reach the saturation of the spectral shift in the experiment, as shown in Figure 5c. The Δ*f* values of the 90 μm *W* MCWM were greater than those of the 249 μm *W* MCWM for all the PAA membrane densities because of two factors. The first factor was the high filling ratio under the same PAA infiltration quantity, which meant that the refractive index of air, 1.0, was replaced by that of a PAA membrane, >1.0, so that the 90 μm *W* MCWM had a greater OPL. Second factor was the sharp modal profile with a high peak intensity (Figure 3e) that effectively interacted with the loaded PAA membranes. However, for the same filling ratio in the FEM calculation, the dip Δ*f* values of the 90 μm *W* MCWM were still higher than those of the 249 μm *W* MCWM, as presented in Figure 5d. This finding showed that the experimental results of the spectral responses in Figure 5c were mostly determined by the second factor, which achieved a strong field–analyte interaction.

**Figure 6:**
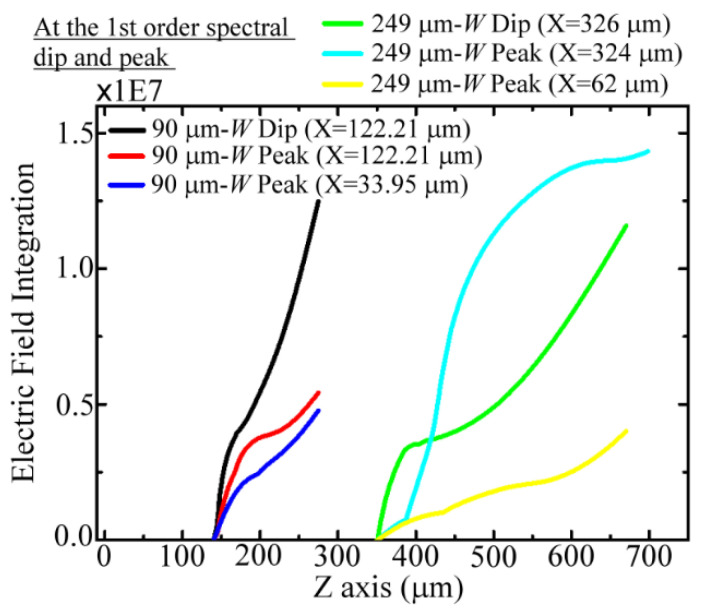
Simulated *Z*-axial E-field integrals of 90 and 249 μm *W* MCWMs at the first-order spectral dips and peaks.

On the basis of the E-field distributions in Figure 3e,f, the integral of the 1D E-field profile with respect to the *Z*-axial position is illustrated in Figure 6, where the integrating ranges are 150–290 and 350–690 μm for the 90 and 249 μm *W* MCWMs, respectively. Figure 6 depicts the accumulated electric field intensity along the Z-direction while the membrane thickness increasingly infiltrated the pores, corresponding to the thickness increase in the *Z*-axial position. For the 249 μm *W* MCWM, the E-field integral of the peak frequency was much greater than that of the dip frequency when the membrane thickness was large enough for *Z* > 419 μm (cyan and green curves, respectively, in Figure 6). The results of the 1D E-field integral for sensing thick membranes thus demonstrated that the peak Δ*f* values were larger than the dip Δ*f* values for sensing PAA membranes with the 249 μm *W* MCWM (Figure 5c). However, the curve trend was inverted as the membrane thickness became thinner than 419 μm (*Z* < 419 μm). The measured dip Δ*f* values approximate to the peak Δ*f* values for a PAA density lower than 10 μg/mm^2^ cannot be interpreted from Figure 5c, because the 1D integral of the E-field was calculated at only one specific *X*-axial position, which was insufficient for reflecting the overall field–analyte interaction strength. To evaluate the interactive strength on a thin membrane of less than 419 μm, the 2D integral of the electric field on the *XY* plane should be considered for each *Z*-axial position.

For the 90 μm *W* MCWM, the measured Δ*f* values of the dip and peak in Figure 5c were similar and rapidly increased below the sample density of 10 μg/mm^2^. The dip and peak Δ*f* values were then saturated at 10 and 25 μg/mm^2^, respectively, to terminate their linearly proportional relation with the PAA membrane density (*ρ*) (Figure 5c). The saturation of the spectral shift was due to the almost complete coverage of the *Z*-axial E-field profile with the PAA membrane thickness (Figure 3e,f). This also meant that the OPLs of the dip and peak frequency waves for the 90 μm *W* MCWM did not change obviously at saturation. The measured results presented in Figure 5c showed that the peak Δ*f* values were obviously greater than the dip Δ*f* values when the PAA membrane density increased above 25 μg/mm^2^. These spectral responses of the 90 μm *W* MCWM could be explained by the 1D E-field integrals, as shown in Figure 6. For the thin membrane thickness with a membrane–air interface at the *Z*-axial position of around 150 μm (Figure 6), the E-field integral of the dip frequency was obviously larger than that of the peak frequency, which agreed with the trend observed between the dip and peak Δ*f* values of the 90 μm *W* MCWM for sensing PAA densities lower than 10 μg/mm^2^ (Figure 5c). As the membrane densities (i.e., thicknesses) gradually increased, the dip Δ*f* value reached saturation first due to the more confined E-field profile of the dip frequency wave (Figure 3a) compared with that of the peak frequency wave (Figure 3c). This means that the integral E-field value in Figure 6 for the dip wave, denoted by a black curve, was much greater than that of the peak wave, denoted by the red and blue curves, at the same *Z*-axial position. When the membrane thickness further increased (*ρ* >10 μg/mm^2^), the dip Δ*f* value could not increase further because the *Z*-axial modal profile range was smaller than the PAA membrane thickness. In contrast, the peak Δ*f* values increased for *ρ* > 10 μg/mm^2^ because of the broadened electric field distribution along the *Z*-axis, where the E-field range extended from the metal surface input side to the PET substrate output side, as shown in Figure 3c. This experimental result, presented as green squares and red triangles in Figure 5c, reveals that thickness sensing should be conducted in the near-field range of the apparently confined E-field profile to gain a sufficiently overlapped integral of field and matter, leading to a high-sensitivity performance. 

#### 3.2.2. Thin-Film Sensing: Refractive Index Detection

**Figure 7:**
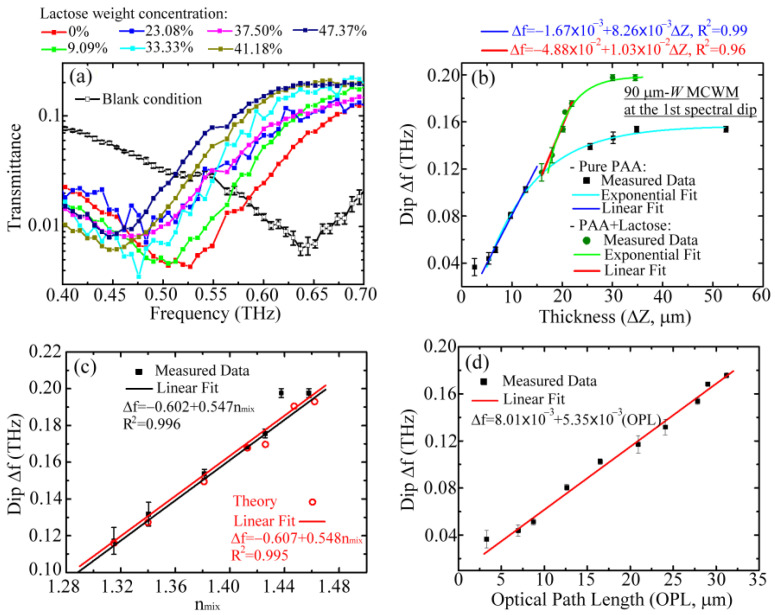
(**a**) Measured transmittance curves of a 90 μm *W* MCWM loaded with lactose-doped PAA membranes for refractive index detection experiment. The measured Δ*f* values of the 90 μm *W* MCWM at the first spectral dip for (**b**) the measured thicknesses Δ*Z*, (**c**) refractive indices, and (**d**) optical path lengths of different lactose-doped PAA membranes.

Figure 7 shows the experimental sensing results of the refractive index using the 90 μm *W* MCWM. To produce a series of membranes with different refractive indices, various weight concentrations of lactose powders were dissolved in the 1 wt% PAA aqueous solution and deposited on the MCWMs via the drop-and-dry process to form lactose-doped PAA membranes. As shown in Figure 7a, the spectral position of the first-order spectral dip was significantly redshifted with increasing lactose dosages. On the basis of the resonance principle of a surface EM mode, this redshift in Figure 7a resulted not only from the increment in the refractive index but also from the increment in the thickness for one lactose-doped PAA membrane. The frequency shift responses to various thicknesses and refractive indices of lactose-doped PAA membranes are respectively illustrated in Figure 7b,c.

Figure 7b summarizes the dip Δ*f* of the 90 μm *W* MCWM in response to various thicknesses (Δ*Z* in Figure 3a–d) of PAA membranes with and without lactose doping, which were respectively obtained from the transmitted spectra in Figure 5a and Figure 7a. The physical thicknesses of the pure and lactose-doped PAA membranes in Figure 7b were measured by an α-step profiler. The doped and undoped pure PAA membranes demonstrated a similar trend in spectral response, with an initially linear proportional relation followed by saturation between the Δ*f* and Δ*Z* parameters. These lactose-doped and pure PAA membrane trends were fit with exponential functions that are illustrated in Figure 7b by the green and cyan curves, respectively. Within the same thickness range of 15–20 μm (Figure 7b), the measured dip Δ*f* values of the lactose-doped PAA membranes exhibited a proportional response and were obviously larger than those of the pure PAA membranes, whose Δ*f*–Δ*Z* relation curve in the 15–20 μm Δ*Z* range reached saturation inversely. Additionally, the measured dip Δ*f* of the 0% lactose membrane was located on the cyan curve of the pure PAA membrane, which represents the thickness sensing results for the pure PAA membranes (Figure 5a). For lactose concentrations above 0%, the corresponding dip Δ*f* values were larger than those of pure PAA membranes above 15 μm. The dip Δ*f* values of the lactose-doped PAA membranes were thus larger than those of the pure PAA membranes even though they had the same physical thicknesses above 15 μm. This finding reveals that the linear response of the Δ*f*–Δ*Z* relation and the high measured Δ*f* values for the doped PAA membranes predominantly originated from the refractive index change induced by the various lactose doping ratios (Figure 7b). According to the slope of the linear fitting curve (the blue line in Figure 7b) and the minimum spectral resolution of 7.32 GHz for our THz-TDS system, the sensitivity and LOD of the thickness sensing could thus be estimated as 8.26 GHz/μm and 886 nm (λ/531), respectively, for pure PAA membrane detection. The minimum thickness measured in the experiment was 5.36 μm, corresponding to λ/88. This also reflects that the scale at which the PAA membrane surface roughness influences the measurement or thickness sensing uncertainty is approximately the micrometer level.

Figure 7c displays the measured and calculated dip Δ*f* values versus various effective refractive indices (*n_mix_*) of lactose-doped PAA membranes, whose dip Δ*f* values were obtained from those of the green circular points in Figure 7b with a thickness range of 15–35 μm. The corresponding *n_mix_* values were estimated as described below. The *n_mix_* of the lactose-doped membranes in the measurement experiments was calculated based on the effective medium theory [40], as shown in Equation (2):

(2) $$n_{mix}^{2}=n_{lactose}^{2}\rho_{lactose}+n_{PAA}^{2}\left(1-\rho_{lactose}\right)$$

where *n_PAA_*, *n_lactose_*, and *ρ_lactose_* are the refractive indices of PAA, lactose, and the volume ratio of lactose for one lactose-doped PAA membrane, respectively. The refractive indices of the lactose and PAA used to calculate the *n_mix_* value in Equation (2) were 1.64 and 1.3, respectively, as measured from lactose tablets and thick PAA films in a THz-TDS system. The simulated dip Δ*f* of the lactose-doped PAA membrane in Figure 7c could be acquired from the FEM and iterative method based on the initial *n_mix_* and depth filling ratios that were obtained from the measured membrane thicknesses in Figure 7b. The measured and calculated results in Figure 7c show that the proportional response between the dip Δ*f* and *n_mix_* occurred in both the experiment and the numerical simulation with a high degree of agreement. For lactose concentrations of 0–37.5% (Figure 7a) or measured thicknesses of 15–20 μm (green circles in Figure 7b), the proportional relation between Δ*f* and *n_mix_* was further linearly fitted, and a coefficient of determination greater than 0.99 (*R*^2^ > 0.99) was found (Figure 7c). The other measured *n_mix_* data (i.e., *n_mix_* = 1.437 and 1.457) for lactose concentrations above 37.5% or measured thicknesses larger than 20 μm were not considered in this linear fitting process. According to the slope of the linear regression for the detection of small amounts of lactose (i.e., *n_mix_* of 1.315–1.425 in Figure 7c) and the measured Δ*f* inaccuracy, the sensitivity and the LOD of the 90 μm *W* MCWM refractive index sensing were 547 GHz/RIU and 0.0134 RIU, respectively.

On the basis of the experimental results of thin-film sensing (Figure 7b,c), the dip Δ*f* was strongly correlated with the optical constant *n_mix_* and the thickness Δ*Z* of the membranes. The dip Δ*f* of the 90 μm *W* MCWM with respect to the membrane OPL, excluding the saturation region, is further summarized in Figure 7d, where the OPL value equals the product of the *n_mix_* and Δ*Z*. That is, the data points in Figure 7d were collected from the data points covered by the red and blue fitting lines in Figure 7b. The dip Δ*f* values proportionally increased with the OPL values, having an extremely high coefficient of determination (R^2^ ~0.99) in the linear regression. On the basis of the linear fitting slopes and the measurement errors of the Δ*f* in Figure 7d, the sensitivity of thin-film sensing, represented in terms of OPL, was estimated as 5.35 THz/RIU∙mm. Based on the OPL sensitivity, the LOD of a high-refractive-index material, such as a semiconductor, could decrease lower than that of a polymeric material with a low refractive index.

#### 3.2.3. Detection of an Inhomogeneous and a Nonuniform Analytes

**Figure 8:**
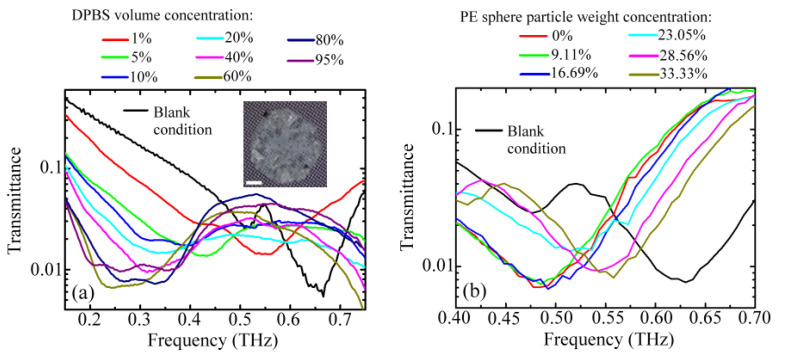
Measured transmission spectral curves of 90 μm *W* MCWM for sensing various amounts of (**a**) salt grains and (**b**) PE sphere particles.

The pure and lactose-doped PAA overlayers used respectively for the thickness and refractive index detections were essentially homogeneous and uniform for the MCWMs. However, when the dielectric membranes become inhomogeneous or nonuniformly distributed analytes, the sensing capability and sensitivity analysis of the MCWMs should be individually specified. In the study, the electrolyte salts and PE microspheres were considered nonuniform and inhomogeneous samples for MCWM sensing, respectively. Figure 8a illustrates the measured transmission spectra of the 90 μm *W* MCWM for sensing various amounts of salt grains, which were prepared from different concentrations of DPBS. The findings showed that the first spectral dip shifted to the low-frequency region compared to that of the blank 90 μm *W* MCWM as the salt particle amount increased. The inset photo of Figure 8a shows the salt grains, obtained from a 40 *v*/*v*% DPBS solution and nonuniformly deposited on the 90 μm *W* MCWM. Figure 8b shows the transmission spectrum of the 90 μm *W* MCWM for different concentrations of PE microparticles mixed in the PAA membrane. The thin PAA polymeric matrix could stably fix the PE sphere particles. However, the dip Δ*f* values were reduced as the concentration of PE microparticles increased, which was opposite to the spectral responses for salts.

**Figure 9:**
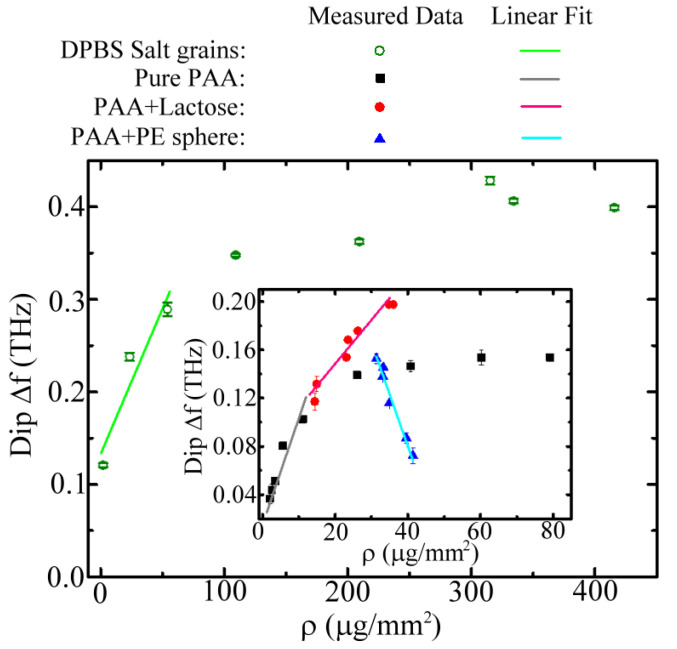
Measured Δ*f* values of 90 μm *W* MCWM at the first spectral dip for depositing different kinds and amounts of analytes.

Figure 9 summarizes the experimental spectral responses of the 90 μm *W* MCWM at the first spectral dip Δ*f* relative to different weight densities of the four kinds of samples, namely the lactose-doped and pure (undoped) PAA membranes, PE microparticles, and salt grains. For the sensing results of the PE sphere particles, the thickness of the deposited PAA matrix was approximately 25 μm, as estimated from the dip Δ*f* values of both 0 wt% PE-particle-doped and pure PAA membranes, which were measured individually in the experiments. That is, the dip Δ*f* value of the first data point of the blue triangles in Figure 9 matched that of the pure PAA membrane with a thickness of 25 μm, as detected in Figure 7b. The 25 μm thick PAA matrix caused the dip Δ*f* value to approach the saturated dip shift of the undoped PAA membrane (black squares of Figure 9). The thickness was sufficient to firmly adhere the PE sphere particles to the metallic surface of the MCWM; however, it was obviously smaller than the PE sphere diameter (34–50 μm). The experimental results showed that the large PE sphere particles scattered the field of the THz surface EM mode on the MCWM surface without driving the THz dispersion of PAA and inevitably reduced the dip Δ*f* value, which was severe at high concentrations of microspheres, as shown in the inset of Figure 9 (blue triangles).

The experimental results of the PE sphere particles (inset of Figure 9) showed that the measured dip Δ*f* within the linear response region ranged from 0.153 to 0.072 THz, corresponding to a maximum frequency variation of 0.081 THz. This result approximated the saturated frequency variation of 60–80 GHz for sensing polystyrene microbeads using e-SRR-based metamaterials [41]. This study demonstrated that the microbeads should be placed precisely in the microgaps of the e-SRR arrays to reach the maximum frequency variation of 80 GHz [41]. The necessity of placing sample particles in critical locations limits the practical applications of this method. Aside from the PE sphere particles, the other three sample types showed proportional and saturated Δ*f* value responses at low and high particle densities. The saturated Δ*f* values for salt sensing were more than two-fold higher than the others (Figure 9) because of the larger refractive indices of the salt grains in the THz frequency. The measured refractive index of the DPBS salt tablets was around 2, which was higher than that of the PAA slab (1.3) and the lactose tablet (1.64). This finding revealed that the sample refractive index had a substantial impact on the first spectral dip shift of the MCWMs.

## 4. Discussion

The sensing performance of the 90 μm *W* MCWM at the first-order spectral dip, represented in terms of the sensitivity and LOD, is summarized in Table 2 for the sensing of four different analytes, namely pure and lactose-doped PAA membranes, electrolyte salts, and a PE microsphere/PAA blend. In the uniform thin-film sensing experiment, the linear spectral responses were mainly caused by changes in the thickness and refractive index of the pure PAA and lactose-doped PAA membranes, respectively. However, for nonuniform sample sensing, e.g., electrolyte salts and PE microsphere/PAA blend, the dip Δ*f* had a proportional relation only to the sample amount, which was represented by the surface weight/molar density. The corresponding weight/molar sensitivities were calculated from the slopes of the linear regressions and are listed in Table 2. The best sensitivity was achieved in the pure PAA membrane sensing experiment, which achieved weight and molar sensitivities of 8.82 × 10^−3^ THz/μg∙mm^2^ and 29.12 GHz/pmole/mm^2^, respectively, with LODs of 732.43 ng/mm^2^ and 221.84 fmole/mm^2^. This result was attributed to the highly viscous PAA polymer, which could easily fill the tiny interstices near the metal gaps formed by the cross-stacked woven wires of the MCWM structure. The PAA-filled tiny interstices had the strongest and most localized THz surface EM mode of the first spectral dip (Figure 3a), thereby facilitating the effective interaction of the samples with the enhanced field to realize a femtomolar-level LOD. 

The 2D planar MHA and MMD sensing performances for thin films and bioanalytes, including the frequency sensitivity (FS) and LOD, are summarized in Table 3 and compared with those of the MCWMs. The parameters related to the FS and LOD included the refractive index and thickness of the analyte and the probe frequency and wavelength of the sensing device, which are denoted as *n*, *h*, *f_0_*, and λ_0_, respectively, in Table 3. The authors of [22] report that the measured thickness and refractive index sensitivities of an MHA with a plasmonic structure for sensing dielectric thin films based on the EOT peak shift were 1 GHz/μm and 128 GHz/RIU, respectively. Using the same EOT effect, the authors of [23] developed polypropylene-substrate-based asymmetric MHAs for sensing photoresist thin films with a sensitivity of 1.54 THz/RIU∙mm. A metallic mesh based on an intensity interrogation at the EOT peak at 0.95 THz was experimentally demonstrated to sense poly(ethylene terephthalate) films with a thickness resolution of 1 μm, corresponding to λ_0_/316 [24]. A similar MMD with an EOT peak at 10 THz, i.e., the middle infrared (IR) region, was successfully demonstrated for sensing 100 nm thick SiO_2_, corresponding to λ_0_/300, based on the frequency interrogation of the Fano-resonant dip [21]. The same MMD scheme and Fano-resonance sensing mechanism have also been demonstrated at 100 THz (i.e., the near IR region) to identify different quantities of concanavalin A protein molecules, achieving an LOD of 10 fmol/mm^2^ [20]. For subwavelength-thick film sensing, the minimum detectable thickness of the IR MMD is approximately 0.1 μm [21] due to the strong field confinement at the metal–air interfaces; nevertheless, the corresponding LOD, ~λ_0_/300, represented by the probe wavelength is inferior to that of the MCWM device, ~λ_0_/531. In summary, the performance of the MCWM surpasses that of planar MHAs and MMDs in thin-film sensing [21,22,23,24] based on the sensitivity and LOD in terms of thickness, refractive index, and OPL. The sensing experiments also proved that the MCWM achieved periodic 3D corrugation of the metal–dielectric composite structure on the XY plane (Figure 1a) to form a strongly confined EM modal distribution with a high peak intensity at the first spectral dip, thus significantly promoting field–matter interaction in the near-field range and improving the sensitivity.

The above sensing results for thickness and refractive index detection using MCWM devices were presented only for dry analytes. For biomolecular analytes with water or biological fluids in their surroundings, the MCWM sensing platform also showed the ability to identify sufficiently thin water-containing biomaterials. The absorption coefficient, refractive index, and penetration depth of liquid water at 0.3 THz are around 130 cm^−1^, 2.7, and 80 μm, respectively [42]. The refractive index of most water-containing bioanalytes is greater than 2 [42], which is approximate to that of DPBS salts. For the sensing scheme of the 90 μm *W* MCWM, the high-index analyte (*n* ≥ 2) shifted the sensing probe frequency of 0.637 THz to 0.2–0.3 THz (Figure 8a) or an even lower THz frequency range. Compared to the high probe frequency for dry analytes, the lower probe frequency for water-containing analytes achieved less water absorption and a deeper water penetration depth [42], consequently promoting the SNR of the sensing device. For the 90 μm *W* MCWM, there was an additional 80% power loss at 0.3 THz due to interfacial reflection loss and water absorption when the liquid water filled the 128 μm thick sample tray (i.e., the 2D thickness value in Table 1) of this sensing device. The sensing performance of the MCWM under this 80% power loss was still adequate based on the 100-SNR of the analyte-loaded MCWM. The high-loss analyte in a 128 μm thick tray filled with water or fluid would broaden the sharp spectral features of the probe frequency and therefore decrease the corresponding Q factor and SNR of the sensing device (Figure 8a). In practical terms, the water-containing bioanalyte with a thickness of less than 80 μm could be identified by the MCWM device with a THz power transmittance > 40%. Compared to the sensing performance for the dry PAA membranes, the sensitivity and LOD of the MCWM sensing device for the detection of high-index and -loss analytes, such as DPBS electrolyte salts, decreased, as shown in Table 2.

## 5. Conclusions

A metal–insulator composite plasmonic structure based on an MCWM was demonstrated in the THz regime and successfully applied for the label-free sensing of a minute amount of thin-film-type and nonuniformly distributed analytes. Two spectral features, namely the resonant dip and peak in the MCWM transmission spectra, were demonstrated to agree well between the measurements and the numerical simulations. The origin of the sharp transmission dips and peaks was validated to strongly correlate with the resonant excitation of the THz surface modes via the aperture (*A_unit_*) and periodicity (*Λ*) of the MCWM unit cell, respectively. The latter was consistent with the EOT phenomenon of planar MHAs. According to the FEM theoretical calculations, the electric field strength and confinement of a 90 μm *W* MCWM at the first-order resonant dip were distinctly higher than those at the first-order EOT resonant peak and those of the 249 μm *W* MCWM. Consequently, the former was adopted in experiments for sensing membranes with different thicknesses and refractive indices. The infiltration of the analytes into the metal gaps near the sinusoidal corrugations of the MCWM, which accumulated the strongest and localized THz surface fields, could effectively raise the absorption cross-section and improve the sensitivity. In the experiment, the highest sensitivities of the MCWM device for thickness and refractive index sensing were 8.26 GHz/μm and 547 GHz/RIU, respectively. The LOD for physical thickness and refractive index were 886 nm (λ/531) and above 5.36 μm for pure PAA membrane sensing and 1.34 × 10^−2^ RIU for lactose-doped PAA membrane sensing, respectively. Both sensing processes were operated at a resonant frequency of 0.64 THz and showed a subwavelength-thick film detection capability. We also conceptually demonstrated the feasibility of quantitatively identifying nonuniformly distributed microparticles, mimicking macro-biomolecules or cells, by integrating analytes into the viscous PAA polymer matrix on the MCWM sensor. The polymeric matrix could fix target analytes within the near-field range to efficiently interact with the probed surface EM field of the MCWM sensor. Based on the optimized MCWM platform, different types and quantities of minute analytes were successfully recognized, and a femtomolar level of molecular concentration sensitivity (221.84 fmole/mm^2^) could be reached for PAA macromolecule detection. This work presents a rapid, inexpensive, and simple analysis method, potentially paving the way for a new generation of label-free microanalysis sensors. 

## Figures and Tables

**Table 1:** Dimensions of two MCWMs in experiments.

	W (μm)	D (μm)	Λ (μm)	Open Ratio (%)
Large-pore MCWM	249	120	738	45
Small-pore MCWM	90	64	308	30

**Table 2:** 90 μm *W* MCWM sensitivity and LOD for sensing different kinds of analytes.

	Pure PAA	PAA + Lactose	Electrolyte Salts	PAA + PE Sphere
Weight sensitivity	8.82 × 10^−3^ THz/μg∙mm^2^	3.64 × 10^−3^ THz/μg∙mm^2^	3.14 × 10^−3^ THz/μg∙mm^2^	−8.4 × 10^−3^ THz/μg∙mm^2^
Molar sensitivity	0.02912 THz/pmole/mm^2^	0.00187 THz/pmole/mm^2^	0.20078 THz/μmole/mm^2^	–
Weight LOD	732.43 ng/mm^2^	2.01 μg/mm^2^	2.36 μg/mm^2^	–
Molar LOD	221.84 fmole/mm^2^	3.91 pmole/mm^2^	36.96 nmole/mm^2^	–

**Table 3:** Sensing performance comparison among the MHA, MMD, and MCWM device schemes.

Reference	*f*_0_ (THz)	*f*_0_ Region	n	h (μm)	FS (Original)	FS = Δ*f*/(n∙h) (GHz/RIU∙mm)	LOD	LOD (λ_0_)
[22]	0.52	THz	1–2	50	128 GHz/RIU	2560	50 μm	1/11
[22]	0.52	THz	1.41	5–50	1 GHz/μm	709	5 μm	1/115
[23]	0.85	THz	2	13	0.8	1540	3 μm	1/118
[24]	0.95	THz	1.7	3–5	–	–	1 μm	1/316
[21]	10	Middle IR	2.5	0.03–0.5	295 GHz/μm	404,000	0.1 μm	1/300
[20]	100	Near IR	–	–	5 GHz/fmol∙mm^−2^	–	10 fmol/mm^2^	–
MCWM (the present work)	0.637	THz	1.3	5–53	8.26 GHz/μm 29.12 × 10^−3^ GHz/fmol∙mm^−2^	5350	0.886 μm; 221.84 fmol/mm^2^	1/531
MCWM (the present work)	0.637	THz	1.32–1.46	16–35	547 GHz/RIU 1.87 × 10^−3^ GHz/fmol∙mm^−2^	5350	1.34 × 10^−^^2^ RIU	–

## Data Availability

Not applicable.
